# *HLA-C* Alleles and Cytomegalovirus Retinitis in Brazilian Patients with AIDS

**DOI:** 10.1155/2018/3830104

**Published:** 2018-09-02

**Authors:** Tânia Gisela Biberg-Salum, Maria de Lourdes Veronese Rodrigues, Ana Paula Morais Fernandes, Neifi Hassan Salum Deghaide, Jayter Silva de Paula, Erick C. Castelli, Celso Teixeira Mendes-Junior, Eduardo Antonio Donadi

**Affiliations:** ^1^Ribeirao Preto Medical School, University of São Paulo, University for Development of Pantanal Region (UNIDERP), São Paulo, SP, Brazil; ^2^Department of Ophthalmology, Otorhinolaryngology and Head and Neck Surgery, Ribeirao Preto Medical School, University of São Paulo, São Paulo, SP, Brazil; ^3^Department of General and Specialized Nursing, Nursing School of Ribeirao Preto, University of São Paulo, São Paulo, SP, Brazil; ^4^Molecular Immunology Laboratory and HLA Hemocentro, Ribeirão Preto Medical School University Hospital, Ribeirão Preto, Brazil; ^5^School of Medicine, Department of Pathology and Molecular Genetics and Bioinformatics Laboratory, São Paulo State University, Botucatu, SP, Brazil; ^6^Departamento de Química, Laboratório de Pesquisas Forenses e Genômicas, Faculdade de Filosofia, Ciências e Letras de Ribeirão Preto, Universidade de São Paulo, 14040-901 Ribeirão Preto, SP, Brazil; ^7^Division of Immunology, Department of Internal Medicine, Ribeirao Preto Medical School, University of São Paulo, São Paulo, SP, Brazil

## Abstract

**Purpose:**

Since cytomegalovirus retinitis (CR) is an important cause of visual impairment among AIDS patients and *HLA-C* alleles have been associated with AIDS disease outcome, we typed *HLA-C* locus in patients with AIDS exhibiting or not CR.

**Methods:**

Three groups of individuals were studied: (i) 49 patients with AIDS and CR (Group I), (ii) 161 patients with AIDS without CR (Group II), and (iii) 202 healthy HIV-negative individuals (Group III). *HLA-C* typing was performed using commercial kits.

**Results:**

The *HLA-C∗*07 allele group was underrepresented in AIDS patients with CR (*P*=0.005) when compared to controls or when compared to AIDS patients without CR (*P*=0.006). The *HLA-C∗*05 allele group was overrepresented in Group II in comparison to Group III (*P*=0.017). The frequency of the *HLA-C∗*16 allele group was increased in Group III in comparison to Group II (*P*=0.004).

**Conclusion:**

The *HLA-C∗*07 allele group conferred protection against the development of CR in Brazilian AIDS patients, whereas the *HLA-C∗*05 and *HLA-C∗*16 allele groups were associated with AIDS susceptibility or protection, respectively.

## 1. Introduction

Cytomegalovirus retinitis (CR) is the most common ocular opportunistic infection in patients with AIDS, often leading to blindness [1]; however, among patients who experience immune recovery after antiretroviral treatment (ART), an important reduction in the incidence of CR has been reported [2]. Despite recent advances in treatment, CR continues to rise challenging diagnostic and therapeutic questions since not all patients respond to ART [3].

Progression to AIDS has been strongly associated with the human leukocyte antigen B (*HLA-B*) alleles, particularly *HLA-B∗*57 and *HLA-B∗*27 allele groups (protection), and *HLA-B∗*35 (susceptibility), due to the restricted recognition of HIV epitopes by cytotoxic immune system cells [4]. An increasing body of evidence has pointed out the role of HLA-C on HIV-1 disease progression. *HLA-C* gene diversity has an impact on HIV infection, particularly a polymorphism located at the promoter region of the *HLA-C* gene (-35C/T), for which the -35C variant has been associated with low viral load and high *HLA-C* mRNA [5].

We have previously reported that susceptibility to CR was associated with *HLA* alleles associated with rapid progression to AIDS, especially *HLA-B35* [6]. To further understand the impact of *HLA-C* polymorphism on CR development among Brazilian patients with AIDS, we evaluated the *HLA-C* allele polymorphism in patients presenting or not CR.

## 2. Materials and Methods

### 2.1. Subjects

An observational case-control study was carried out, using DNA samples obtained from patients with AIDS and from healthy individuals, stored in a DNA Sample Bank (Banco de Amostras do Núcleo de Pesquisa em Imunogenética (BANPI)), approved by the local Ethics Committee #7581/2007). All the 210 patients with AIDS were regularly registered at the Brazilian Ministry of Health and were followed up at the University Hospital of the Ribeirão Preto Medical School, the officially recognized hospital to follow-up these patients.

Group I consisted of DNA samples from 49 patients with AIDS presenting CR (38 men aged 21–77 years, median* *=* *35 years), collected regardless of the evolutionary phase or the antiviral treatment of retinochorioiditis. CR was diagnosed by at least two experienced observers, and concordance between them was considered to be a *sine qua non* condition for patient inclusion. Clinical evaluation was performed by indirect binocular ophthalmoscopy, using a Heine Omega 100® ophthalmoscope (Herrsching, Germany), and a 20* *diopter Nikon® lens (Melville, USA) under mydriasis produced by the local use of 1% tropicamide (Mydriacyl®, Alcon, São Paulo, Brazil). The CD4^+^ cell count ranged from 1 to 368* *cells/mm^3^ (median* *=* *31), and the viral load ranged from 400 to 8,500,000* *copies/mL (median* *=* *85,000). Only 19 (43.2%) of these patients were on antiretroviral therapy. The time since the diagnosis of AIDS ranged from 1–105 months (median* *=* *22 months), and the number of systemic opportunistic infections preceding CR ranged from 0–6 (median* *=* *3). The most frequent infections included candidiasis, neurotoxoplasmosis, pneumocystosis, and neurocryptococcosis.

Group II consisted of DNA samples obtained from 161 patients with AIDS (102 men aged 21–62 years, median* *=* *37 years), randomly selected after performing the ophthalmologic examination excluding the presence of CR and exhibiting no changes of the choroid and retina or any other doubtful retinal diagnosis. The CD4^+^ cell count ranged from 4 to 1164 cells/mm^3^ (median* *=* *588), and the viral load ranged from 80 to 2,000,000* *copies/mL (median* *=* *85,000). Time since the diagnosis of AIDS ranged from 1–108 months (median* *=* *20 months), and these patients experienced 0–8 systemic previous opportunistic infections (median* *=* *2), the most frequent being candidiasis, neurotoxoplasmosis, tuberculosis, and neurocryptococcosis.

Group III consisted of DNA samples from 202 healthy individuals (146 men aged 18–59 years, median* *=* *33 years), exhibiting negative serology for HIV-1.

### 2.2. *HLA-C* Typing

Patient DNA was obtained from peripheral venous blood collected during the patient follow-up at Unit for the Treatment of Infectious Diseases (UETDI), and healthy control DNA was obtained from bone marrow donors.

*HLA-C* typing was carried at the Laboratory of Molecular Immunology of HCFMRP-USP, using a commercial kit (Micro SSP DNA Typing Tray, One Lambda, Canoga Park, CA).

### 2.3. Statistical Analyses

Intergroup differences were determined by the two-tailed Fisher exact test, with calculation of the odds ratio (OR) and 95% confidence interval (95% CI). The level of significance was set at *P* ≤ 0.05 in all analyses. In addition, the etiologic fraction (EF) was calculated to determine how much each allele, genotype, or haplotype contributed to susceptibility to CR. The preventive fraction (PF), which estimates how much these same three factors contribute to protection against CR, was also calculated.

## 3. Results

The *HLA-C∗*07 allele group was underrepresented in Group I compared to Group II (OR: 0.4233, 95% CI: 0.2246–0.9782, *P*=0.006), yielding a PF of 0.1531 (Table 1).

When the *HLA-C* allele frequencies were compared between Groups I and III, an underrepresentation of the *HLA-C∗*07 allele group was also observed for Group I (OR: 0.4192, 95% CI: 0.2246–0.7822, *P*=0.005), conferring a PF of 0.1553 (Table 2).

The *HLA-C∗*05 allele group was overrepresented in Group II in comparison to Group III (OR: 2.2045, 95% CI: 1.1665–4.1665, *P*=0.017), conferring an EF of 0.0455. The frequency of the *HLA-C∗*16 allele group was increased in Group III in comparison to Group II (OR: 0.3580, 95% CI: 0.1737–0.7380, *P*=0.004), exhibiting a PF of 0.0524 (Table 3).

## 4. Discussion

It has been recognized that CR is a condition leading to reduced visual acuity and blindness in patients with AIDS patients; however, with the advent of the more effective antiretroviral therapy, a reduction in the frequency and in the severity of CR has been observed. Notwithstanding, in less favored regions, due to the lack of treatment or incorrect use of medications, or even in more privileged regions due to immunosuppression for organ transplantation, CR continues to deserve special attention.

In the present study, the *HLA-C∗*07 allele group was underrepresented in AIDS patients with CR, when compared to patients with AIDS without CR and when compared to healthy controls, indicating that the allele group is a protective factor against CR development. Considering that CD4 cell counts observed for patients with AIDS exhibiting CR (1 to 368* *cells/mm^3^, median* *=* *31) were very different from patients with AIDS without CR (4–1164* *cells/mm^3^, median* *=* *588), one may argue that CD4 cell count could be a confounding variable. To circumvent this problem, we compared patients with CR with those without CR, stratified according to the CD4 cell counts, that is, (i) patients with AIDS without CR (exhibiting CD4* *≤* *800* *cells/mm^3^; 4 to 778* *cells/mm^3^, median* *=* *215) versus patients with AIDS and CR (exhibiting CD4* *≤* *368* *cells/mm^3^; 1 to 368* *cells/mm^3^, median* *=* *31) and (ii) patients with AIDS without CR (exhibiting CD4* *≤* *100 cells/mm^3^; 4 to 99* *cells/mm^3^, median* *=* *49) versus patients with AIDS and CR (exhibiting CD4* *≤* *100* *cells/mm^3^; 1 to 90* *cells/mm^3^, median* *=* *29). The first comparison revealed that the *HLA-C∗*07 allele group was overrepresented in patients with AIDS without CR (*P*=0.0163), and the second comparison showed that the HLA-C*∗*07 allele group was also overrepresented in patients with AIDS without CR (*P*=0.0298), indicating that the number of CD4 cells counts did not bias the results, and the *HLA-C∗*07 allele group did protect against CR development in patients with AIDS.

*HLA-C* alleles have been associated with protection against AIDS development, particularly for patients exhibiting the -35C allele upstream of the *HLA-C* gene, which has been reported to be associated with high cellular expression of HLA-C molecules and protection against HIV [7, 8]. Notably, many alleles of the *HLA-C∗*07 group have been associated with the -35T allele that contributes to low HLA-C expression and relatively high risk of disease progression [9]. Then, according to the -35CT dimorphism hypothesis, one should expect that an increased frequency of *HLA-C∗*07 alleles is associated with the -35T upstream allele, a fact that was not observed in the present study. Although the -35CT hypothesis is very attractive to explain AIDS outcome due to the immune modulatory role of the *HLA-C* molecule, the complete structure of the *HLA-C* gene (regulatory and coding regions) has not been completely studied. Further studies encompassing larger groups of patients with AIDS exhibiting CR as well as the study of the complete *HLA-C* gene could unravel the role of the gene in the susceptibility to CR. Besides the association with CR, the frequency of the *HLA-C∗*05 allele group was increased in patients with AIDS, irrespective of the presence or not of CR, indicating that this allele group is associated with AIDS susceptibility. On the contrary, patients with AIDS exhibited a reduced frequency of the *HLA-C∗*16 allele group, indicating a protective factor against the AIDS development.

*HLA* molecules have a relevant role on the control of HIV infection. Classic examples are the association of *HLA-B∗*57 and *HLA-B∗*35 with a slower or faster progression of the disease, respectively [10]. Regarding the HLA-C molecules, Silva et al. [11] reported that the *HLA-B∗*04, *-B∗*57, and *-C∗*18 alleles were associated with a low viral load in HIV-infected Brazilian patients. The present results expand the knowledge about the impact of *HLA-C* alleles on CR susceptibility; however, the major limitation of this cross-sectional study is the number of patients with AIDS exhibiting CR, a condition that has been drastically decreased due to the availability of ART.

## 5. Conclusion

The *HLA-C∗*07 allele conferred protection against the development of CR in Brazilian patients with AIDS, whereas *HLA-C∗*05 and *HLA-C∗*16 allele groups were associated with susceptibility/protection against AIDS development. Considering that the etiologic and preventive fractions conferred by these alleles were low, one may assume that many other genes are implicated on disease development.

## Figures and Tables

**Table 1:** *HLA-C* allele frequencies compared between Groups I (patients with AIDS and CR) and II (patients with AIDS without CR).

Allele	Group I (%)	*N*	Group II (%)	*N*	OR	Preventive fraction	*P* value
*HLA-C∗*01	5.10	5	1.55	5			
*HLA-C∗*02	5.10	5	3.10	10			
*HLA-C∗*03	9.18	9	10.24	33			
*HLA-C∗*04	18.36	18	16.45	53			
*HLA-C∗*05	6.12	6	8.07	26			
*HLA-C∗*06	8.16	8	6.52	21			
*HLA-C∗*07	13.30	13	22.67	73	0.4233	0.1531	0.0063
*HLA-C∗*08	2.04	2	3.10	10			
*HLA-C∗*10	0	0	0.31	1			
*HLA-C∗*12	6.12	6	5.27	17			
*HLA-C∗*13	0	0	0.31	1			
*HLA-C∗*14	0	0	1.86	6			
*HLA-C∗*15	5.1	5	3.41	11			
*HLA-C∗*16	7.14	77	3.10	10			
*HLA-C∗*17	5.1	5	3.41	11			
*HLA-C∗*18	0.0	0	1.55	5			

*HLA-C *=* *major histocompatibility complex, class I, C; OR = odds ratio.

**Table 2:** *HLA-C* allele frequencies compared between Groups I (patients with AIDS and CR) and III (healthy HIV-negative individuals).

Allele	Group I (%)	Group III (%)	*N*	OR	Preventive fraction	*P* value
*HLA-C∗*01	5.1	1.73	7			
*HLA-C∗*02	5.1	5.70	23			
*HLA-C∗*03	11.2	6.93	28			
*HLA-C∗*04	22.4	15.33	62			
*HLA-C∗*05	7.1	3.96	16			
*HLA-C∗*06	9.2	9.90	40			
*HLA-C∗*07	13.3	26.73	106	0.4192	0.1553	0.0054
*HLA-C∗*08	2.0	5.70	23			
*HLA-C∗*10	0.0	0.0	0			
*HLA-C∗*12	6.1	6.43	26			
*HLA-C∗*13	0.0	0.0	0			
*HLA-C∗*14	0.0	2.22	9			
*HLA-C∗*15	6.1	3.48	14			
*HLA-C∗*16	7.1	8.17	33			
*HLA-C∗*17	5.1	2.97	13			
*HLA-C∗*18	0.0	0.74	4			

*HLA-C *=* *major histocompatibility complex, class I, C; OR = odds ratio.

**Table 3:** *HLA-C* allele frequencies compared between Groups II (patients with AIDS without CR) and III (healthy HIV-negative individuals).

Allele	Group II (%)	Group III (%)	Etiologic fraction	OR	Preventive fraction	*P* value
*HLA-C∗*01	1.5	1.7				
*HLA-C∗*02	3.4	5.7				
*HLA-C∗*03	10.8	6.9				
*HLA-C∗*04	17.6	15.3				
*HLA-C∗*05	8.3	4.0	0.0455	2.2045		0.0169
*HLA-C∗*06	7.7	9.9				
*HLA-C∗*07	26.5	26.7				
*HLA-C∗*08	3.4	5.7				
*HLA-C∗*10	0.3	0.0				
*HLA-C∗*12	5.9	6.4				
*HLA-C∗*13	0.3	0.0				
*HLA-C∗*14	1.9	2.2				
*HLA-C∗*15	3.7	3.5				
*HLA-C∗*16	3.1	8.2		0.3580	0.0524	0.0041
*HLA-C∗*17	4.0	3.0				
*HLA-C∗*18	1.5	0.7				

*HLA-C *=* *major histocompatibility complex, class I, C; OR = odds ratio.

## Data Availability

The data used to support the findings of this study are available from the corresponding author upon request.
